# Probiotic Consortia and Their Metabolites Ameliorate the Symptoms of Inflammatory Bowel Diseases in a Colitis Mouse Model

**DOI:** 10.1128/spectrum.00657-22

**Published:** 2022-06-22

**Authors:** Limin Xu, Bingdong Liu, Liujing Huang, Ze Li, Yanbo Cheng, Ye Tian, Guihua Pan, Huijun Li, Yinlan Xu, Weidong Wu, Zongbin Cui, Liwei Xie

**Affiliations:** a School of Public Health, Xinxiang Medical University, Xinxiang, China; b State Key Laboratory of Applied Microbiology Southern China, Guangdong Provincial Key Laboratory of Microbial Culture Collection and Application, Guangdong Open Laboratory of Applied Microbiology, Institute of Microbiology, Guangdong Academy of Sciences, Guangzhou, China; c Zhujiang Hospital, Southern Medical University, Guangzhou, China; China Agricultural University

**Keywords:** inflammatory bowel disease, probiotic consortia, metabolites, *Lactobacillus*

## Abstract

Inflammatory bowel disease (IBD) has become a global public health problem. Although the pathogenesis of the disease is unknown, a potential association between the gut microbiota and inflammatory signatures has been established. Probiotics, especially *Lactobacillus* or *Bifidobacterium*, are orally taken as food supplements or microbial drugs by patients with IBD or gastrointestinal disorders due to their safety, efficacy, and power to restore the gut microenvironment. In the current study, we investigated the comprehensive effects of probiotic bacterial consortia consisting of Lactobacillus reuteri, Lactobacillus gasseri, Lactobacillus acidophilus (*Lactobacillus* spp.), and Bifidobacterium lactis (*Bifidobacterium* spp.) or their metabolites in a dextran sodium sulfate (DSS)-induced colitis mouse model. Our data demonstrate that probiotic consortia not only ameliorate the disease phenotype but also restore the composition and structure of the gut microbiota. Moreover, the effect of probiotic consortia is better than that of any single probiotic strain. The results also demonstrate that mixed fermentation metabolites are capable of ameliorating the symptoms of gut inflammation. However, the administration of metabolites is not as effective as probiotic consortia with respect to phenotypic characteristics, such as body weight, disease activity index (DAI), and histological score. In addition, mixed metabolites led only to changes in intestinal flora composition. In summary, probiotic consortia and metabolites could exert protective roles in the DSS-induced colitis mouse model by reducing inflammation and regulating microbial dysbiosis. These findings from the current study provide support for the development of probiotic-based microbial products as an alternative therapeutic strategy for IBD.

**IMPORTANCE** IBD is a chronic nonspecific inflammatory disease. IBD is characterized by a wide range of lesions, often involving the entire colon, and is characterized mainly by ulcers and erosions of the colonic mucosa. In the present study, we investigated the efficacy of probiotics on the recovery of gut inflammation and the restoration of gut microecology. We demonstrate that probiotic consortia have a superior effect in inhibiting inflammation and accelerating recovery compared with the effects observed in the control group or groups administered with a single strain. These results support the utilization of probiotic consortia as an alternative therapeutic approach to treat IBD.

## INTRODUCTION

Inflammatory bowel disease (IBD) is a chronic nonspecific inflammatory disease that occurs in the gastrointestinal tract and includes Crohn's disease and ulcerative colitis (UC) (1). IBD manifests as a wide range of lesions, often extending to the entire colon, and is characterized mainly by ulcers and erosions of the colonic mucosa (2). Most of the clinical symptoms are abdominal pain, diarrhea, bloody stools, and weight loss (3). IBD is a recurrent disease that is difficult to cure and carries a high risk of progression to colorectal cancer (CRC) (4, 5). In recent years, the incidence of IBD has increased at an accelerated rate in newly industrialized countries, increasing the disease burden and impairing the quality of life (6–8). Although the exact etiology of IBD remains unclear, there is considerable evidence that the disease is the result of a combination of genetic, environmental, and dietary factors. At the same time, persistent gut infection, gut microbial imbalance, intestinal mucosal immune regulation, and abnormal gut barrier function are also related to the occurrence and development of IBD (9–11). Medical treatment of IBD focuses on controlling inflammation and preventing disease progression to induce remission. Conventional nontargeted therapies, such as aminosalicylates, glucocorticoids, and immunomodulators, are usually accompanied by strong side effects and poor patient compliance (12). Therefore, targeted biologic therapies have gained more attention and have provided more favorable options for the treatment of IBD (13). For example, anti-tumor necrosis factor (anti-TNF) therapy neutralizes proinflammatory cytokines (14), anti-interleukin 12-p40 (IL-12p40) (targeting the common subunit of IL-12 and IL-23) therapy regulates polarized effector T-cell responses (15), anti-α4β7 integrin therapy reduces proinflammatory cell migration (16), and Janus kinase inhibitors inhibit cytokine signaling in mucosal immune cells (17).

In addition to biologics and targeted small-molecule drugs, traditional Chinese medicine plays an active auxiliary role in the treatment of IBD due to its safety and efficacy. Kuijieling alleviates experimental colitis by regulating the structure and function of gut microbes and modulating the differentiation of Treg and Th17 cells (18, 19). A retrospective study revealed that Chinese herbs such as Plantago ovata, Curcuma longa, and Andrographis paniculata exert their therapeutic benefits through different mechanisms, including immunomodulation, antioxidant activity, and antiplatelet activity (20). In addition, the appropriate amount of micronutrient supplementation is important for the treatment and recovery of IBD (21, 22). More than half of IBD patients have micronutrient deficiency symptoms, e.g., iron deficiency, and the prevalence of anemia in IBD was reported to be 6 to 74% (22, 23). Studies have shown that the host iron-sensing mechanism is closely associated with, and regulated by, the gut microbiota (24). For example, the gut microbial metabolite reuterin can effectively modulate iron homeostasis by inhibiting hypoxia-inducible factor 2α (HIF-2α) function (25). Apart from maintaining systemic iron homeostasis, the gut microbiota is also crucial for maintaining gut health. A group of health-beneficial bacteria, such as Lactobacillus reuteri, was reported to prevent CRC by altering the redox balance in a mouse model (26).

Numerous data have demonstrated that the composition and function of the gut microbiota are key factors affecting the progression of IBD. Dysbiosis of the gut microbiota can lead to damage to the gut barrier and cause intestinal inflammation (27). Dysbiosis in patients with IBD is often accompanied by an increase in pathogenic bacteria and a decrease in beneficial bacteria (28). For example, members of the phylum *Proteobacteria*, such as *Enterobacteriaceae*, particularly certain strains of adherent-invasive Escherichia coli (AIEC), are typically increased in IBD patients relative to healthy individuals (29). In contrast, IBD patients show reduced levels of major short-chain fatty acid (SCFA)-producing bacteria, such as Faecalibacterium prausnitzii (30). SCFAs have been shown to regulate G protein-coupled receptors in the immune response to maintain the intestinal epithelial barrier and prevent intestinal inflammation (31). The use of gut flora and their metabolites, such as with fecal microbiota transplantation (FMT), to treat IBD is also becoming a viable option (32). It is well known that the two main genera of probiotics, *Bifidobacterium* and *Lactobacillus*, have also been reported to have potential benefits in the improvement of IBD and colon cancer symptoms due to their capacity to produce SCFAs (30). In addition, *Lactobacillus* and *Bifidobacterium* can reestablish intestinal symbiosis and protect the intestinal mucosa by increasing the adhesion of healthy probiotics to the intestinal mucosa, inhibiting pathogens from adhering to the mucosal surface, competitively eliminating pathogenic microorganisms, producing antibacterial substances, and regulating immune function (33). For example, L. reuteri converts glycerol into a spectrum of antimicrobial compounds, e.g., reuterin, which suppresses the growth of pathogens and other microorganisms (34). Importantly, L. reuteri R2lc also produces other antimicrobial metabolites, such as polyketides, and activates aryl hydrocarbon receptor (AhR), which is associated with intestinal inflammation (35, 36). Lactobacillus gasseri protects the intestine from dextran sodium sulfate (DSS)-induced colitis and maintains immune homeostasis by increasing the antioxidant response (37). The combination of Lactobacillus acidophilus and Bifidobacterium animalis subsp*. lactis* exerts anti-inflammatory effects by modulating TLR2-mediated activation of the NF-κB and MAPK signaling pathways (38). Additionally, a double-blind, placebo-controlled study demonstrated that the probiotic VSL#3 containing eight species (four strains of Lactobacilli, three strains of Bifidobacteria, and one strain of Streptococcus thermophilus) was safe and effective in improving the clinical outcome by reducing symptoms and improving the endoscopic appearance of the colonic mucosa in patients with UC (39). Although these studies have demonstrated that L. reuteri, L. gasseri, L. acidophilus, and Bifidobacterium lactis have positive effects on the host, the comprehensive effects of these four probiotics on DSS-induced colitis have not been clearly evaluated, and their underlying therapeutic application in IBD still requires additional exploration.

Therefore, in the present study, we sought to determine the combinational effects of probiotic strains L. reuteri, L. gasseri, L. acidophilus, and *B. lactis* on a rodent colitis model and to investigate how probiotic consortia improve the gut ecosystem by modulating the microbial structure, composition, and function to protect against gut inflammation.

## RESULTS

### The probiotic consortia alleviated the pathological symptoms of DSS-induced experimental colitis.

**FIG 1:**
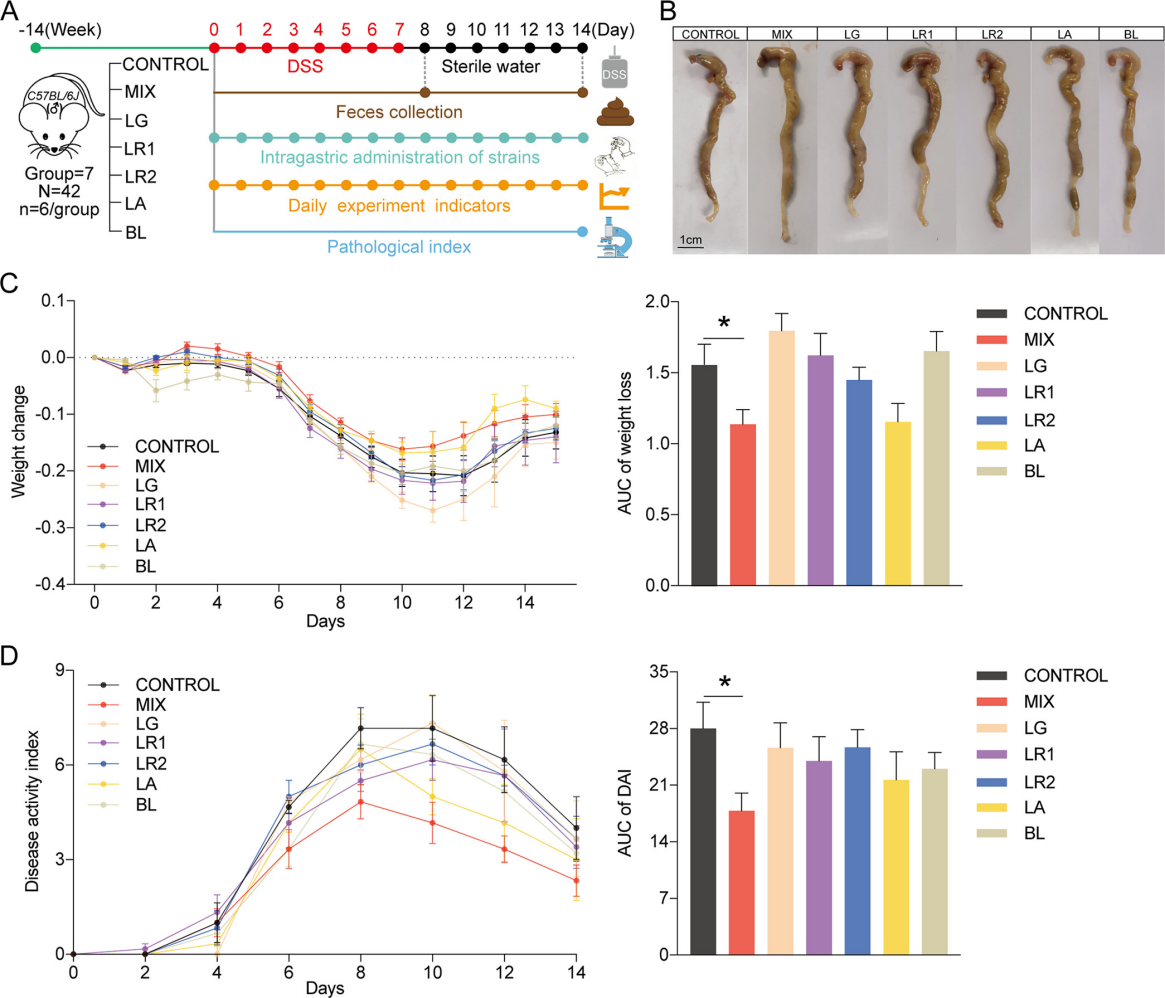
Probiotic consortia attenuate the phenotype of DSS-induced experimental colitis. (A) Schematic of the experimental timeline (CONTROL, control group; MIX, probiotic consortia; LR1, Lactobacillus reuteri PLBK1 group; LR2, Lactobacillus reuteri PLBK2 group; LG, Lactobacillus gasseri PLBK3 group; LA, Lactobacillus acidophilus PLBK4 group; BL, Bifidobacterium lactis PLBK5 group). (B) Representative images of colons for each group. (C) Weight loss and area under the curve (AUC) of weight loss for seven groups (*n* = 6). (D) Disease activity index (DAI) and AUC of DAI (*n* = 6). Statistics were calculated with one-way ANOVA. *, *P < *0.05. Data are presented as the mean ± SEM.

To understand the effect of the five probiotic strains on colitis, a DSS-induced colitis mouse model was established with 14-week-old C57BL/6*J* mice. The colitis mouse model was established by adding 2.5% DSS in sterile water for 7 days, followed by a 7-day washout period. The mice assigned to the experimental group were administered a single probiotic or probiotic consortia via gavage, while the control group was fed phosphate-buffered saline (PBS) containing 30% glycerol. Throughout the entire experimental procedure, experimental indicators such as body weight, changes in feces consistency, and bloody feces were recorded daily. Feces were collected on days 8 and 14 for gut microbiota analysis via 16S rRNA gene sequencing, and the colonic tissue was collected after the mice were sacrificed (Fig. 1A). Compared with the control group, the probiotic consortia group alleviated the shortened colon caused by DSS (Fig. 1B), even though there was no significant difference (see Fig. S1A in the supplemental material). In addition, the probiotic consortia group had a moderate weight change curve. The administration of the probiotic consortia significantly reduced weight loss compared with the control group (*P = *0.038) (Fig. 1C). The probiotic consortia group also demonstrated a significantly lower disease activity index (DAI) score than the control group (*P = *0.027) (Fig. 1D). However, the effects of the single probiotic strain on the rate of weight loss and DAI score were not as significant as those of the probiotic consortia.

### The probiotic consortia restored inflammatory damage to the colonic mucosa.

**FIG 2:**
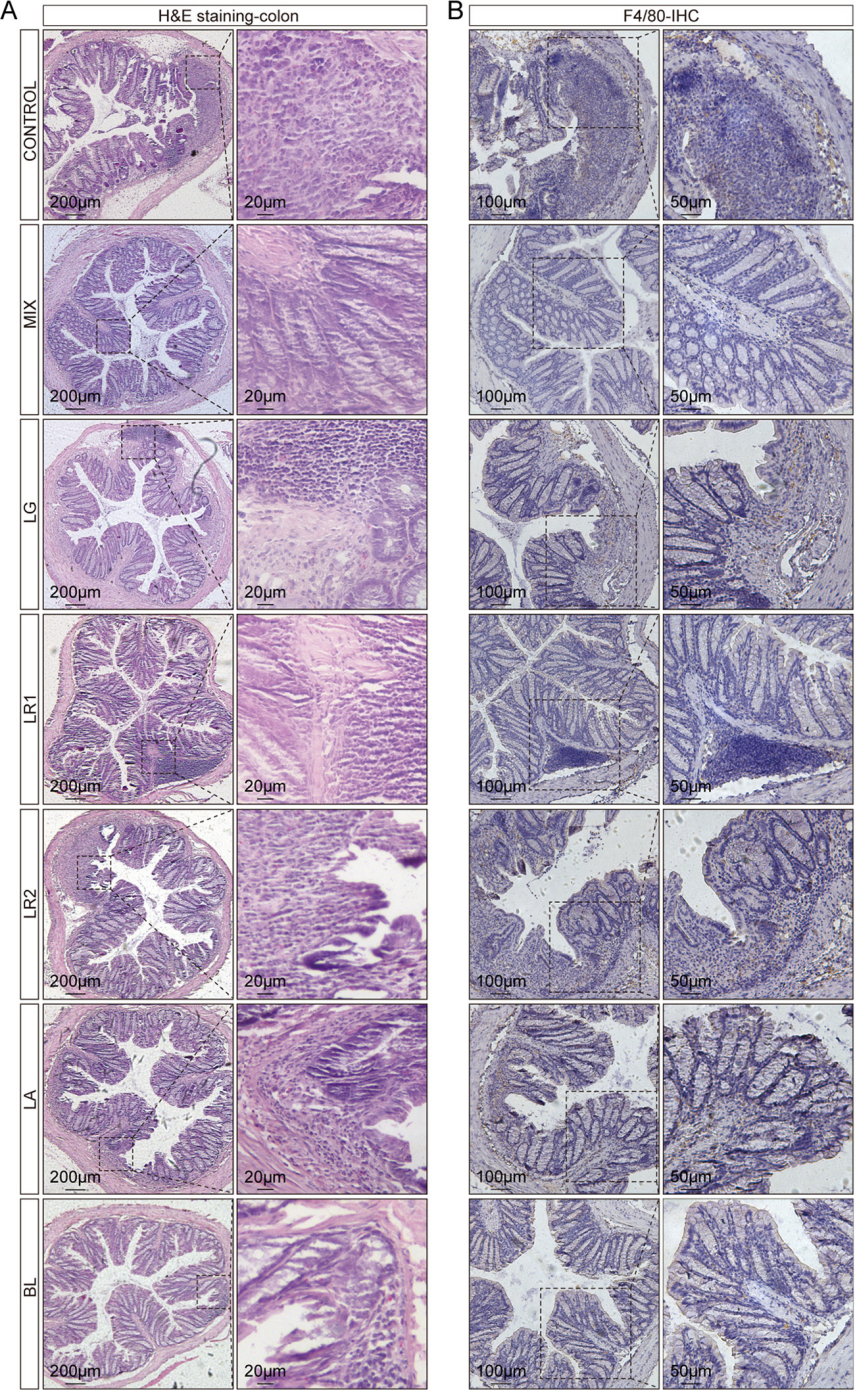
Probiotic consortia protect colon structures and inhibit macrophage infiltration in a DSS-induced colitis mouse model. (A) Representative images of H&E staining. (B) Representative images of F4/80 immunohistochemistry (IHC).

Hematoxylin and eosin (H&E) staining of colon sections was performed to evaluate colonic mucosal injury based on histological analysis. In the control group, the transverse colonic sections exhibited an altered luminal architecture and barrier, manifesting as damage to the mucosal layer, loss of crypts, and infiltration of inflammatory cells (Fig. 2A). In contrast, probiotic consortia treatment yielded protective effects on the colonic mucosa, as indicated by the notably intact crypts and less inflammatory cell infiltration. Furthermore, histological examination of colon sections in the probiotic consortia group revealed markedly lower cumulative scores than those of the control group (Fig. S1B). In addition, even if inflammation could still be observed in the single-strain groups, most of the crypt structures were normal, especially in the Lactobacillus acidophilus PLBK4 (LA) group, which had a lower histology score than the control group (Fig. S1B). Infiltrations of macrophages have been identified as markers of colitis severity (40, 41). To further study the influence of probiotic strains on the colitis mouse model, immunohistochemistry (IHC) of F4/80 was performed to detect inflammation with macrophage infiltration. Consistent with the H&E staining, the level of F4/80 was reduced by the probiotic consortia compared with that of the control group (Fig. 2B).

### The probiotic consortia modulated the structure of gut microbiota in a DSS-induced colitis mouse model.

**FIG 3:**
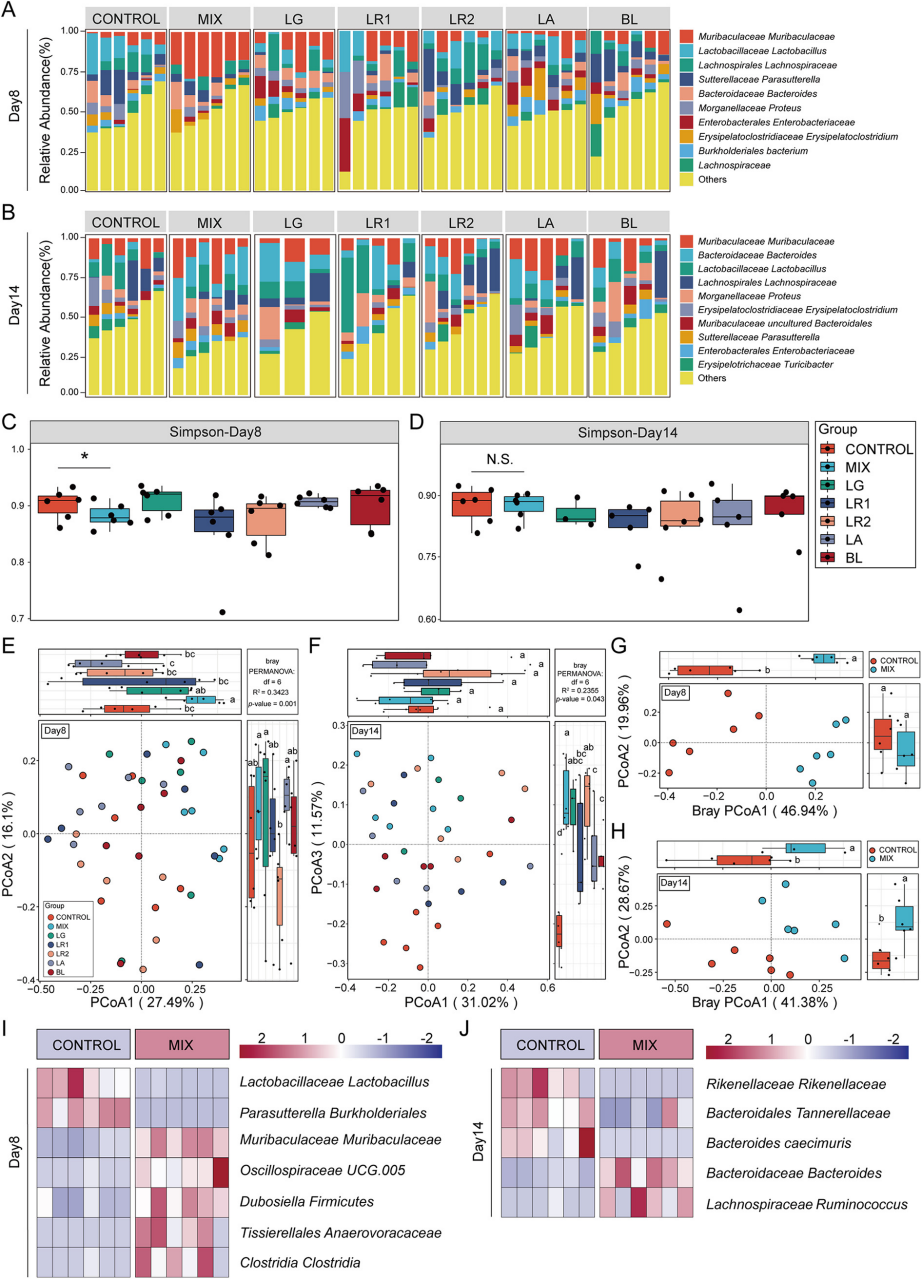
Probiotic consortia restore the composition of the gut microbiota. (A and B) Structure plot of the top 10 abundances at the species level in seven groups on day 8 or 14. Samples were ranked according to the increase in the relative abundance of the species most abundant in each group. (C and D) α-Diversity is represented by the box plot of the Simpson index on day 8 or 14. Statistics were calculated with a two-tailed Student's *t* test. N.S., not significant; *, *P < *0.01. (E and F) The PCoA of β-diversity based on species-level microbiota as assessed by a Bray-Curtis matrix between seven groups on day 8 or 14. Different letters indicate statistical differences (*P < *0.05) between the groups. (G and H) The PCoA of β-diversity based on species-level microbiota as assessed by a Bray-Curtis matrix between the probiotic consortia and control groups on day 8 or 14. (I and J) A heat map indicates the bacterial genus with significant differences (*P < *0.05) between the probiotic consortia and control groups on day 8 or 14.

Colitis is strongly associated with alterations in the composition and structure of the gut microbiota (42). To investigate the effect of probiotic strains in modulating the gut microbiota in a DSS-induced colitis mouse model, we performed 16S rRNA sequencing of fecal samples collected on days 8 and 14 upon DSS treatment. Rarefaction analysis showed that the sequencing depth was sufficient to capture the majority of gene diversity (Table S3) (43). The composition of the gut microbiota of the seven groups varied at the species level in different experimental periods (Fig. 3A and B). Meanwhile, the α-diversity values (Simpson index) were assessed among the seven groups at the species level on days 8 and 14. It was shown that on day 8, the Simpson index of the probiotic consortia group was significantly different from that of the control group but not for the other single-strain-treated groups (Fig. 3C), while the difference between the control and probiotic consortia group did not exist on day 14 (Simpson index) (Fig. 3D). These results demonstrated that probiotic consortia intervention exerts a strong influence on the composition and diversity of microbiota during the persistent stage of inflammation to accelerate recovery. Furthermore, β-diversity with principal-coordinate analysis (PCoA) using the Bray-Curtis distance metric demonstrated that only the gut microbiota of the control group and the probiotic consortia group were clearly separated into two clusters (Fig. 3E and G), implying a significantly altered microbial structure in the probiotic consortia group at the species level on day 8. It is worth noting that on day 14, the PCoA plot of the seven groups was distinct from that of the control group (Fig. 3F and H). From the diversity and structure results of the gut microbiota, it was obvious that the probiotic consortia treatment exerted a strong modulatory effect on the gut microbiota during the persistent and recovery phases of gut inflammation (Fig. S1C). Indeed, the probiotic consortia group also revealed distinct microbial compositions during the recovery phase of gut inflammation, e.g., *Muribaculaceae Muribaculaceae* and *Oscillospirales* UCG.005 on day 8 and *Bacteroidaceae Bacteroides* and *Lachnospiraceae Ruminococcus* on day 14 (Fig. 3I and J, Supplementary file 1 Tax_Info).

### The gut microbiota correlated with the phenotype of the colitis mouse model.

The relationship between microbial dysbiosis and IBD is complex. The gut microbiota plays a key pathogenic role in IBD, while chronic inflammation also contributes to dysbiosis via alteration of the oxidative and metabolic environment of the gut (27). In our study, the results revealed differences in disease phenotypic data (e.g., DAI, colon length, and histological score) and microbial indexes (e.g., α- and β-diversity, relative abundance) in the colitis mouse model between groups. Next, we utilized redundancy analysis (RDA) to determine the correlation between flora and phenotypic data. The results obtained from the permutation test revealed a strong correlation between core microbial and phenotypic data in seven groups at the species level (Fig. S2A, model *P* value, 0.003; Fig. S2B, model *P* value, 0.025). Among them, the core microbial data at the species level were correlated with the phenotypic data (weight, DAI, blood in feces, and stool consistency) in samples from seven groups to a degree of 52.68% (Fig. S2A). After 999 permutation tests, *Bacteroidaceae Bacteroides* (V4), *Sutterellaceae Parasutterella* (V9), *Parasutterella Burkholderiales* (V17), *Muribaculaceae Bacteroidales* (V36), *Firmicutes Bacilli* (V34), and Mucispirillum schaedleri (V42) had statistically significant constraints on the arrangement of the phenotype data. In addition, the core microbial data at the species level were 89.13% correlated with the colon length and history score (Fig. S2B). Moreover, *Muribaculaceae Muribaculaceae* (V8), *Enterobacteriaceae* Klebsiella (V39), *Bacteroidales Muribaculaceae* (V12), and Mucispirillum schaedleri (V42) had a significant effect on the phenotype data.

### Identification of key microbial biomarkers during the recovery phase of gut inflammation.

**FIG 4:**
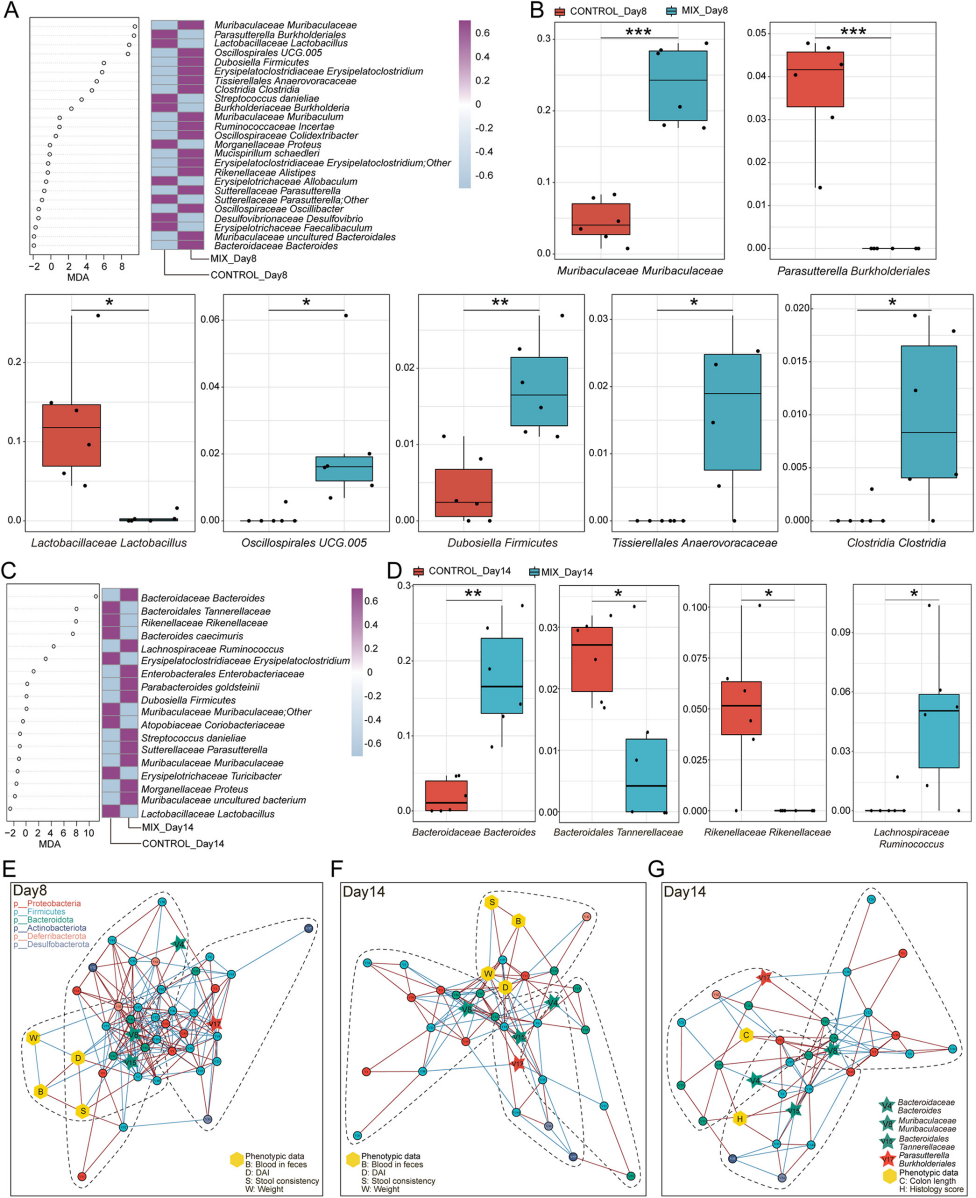
Identification of signature gut microbiota in colitis mouse model by random forest. (A) Mean decrease accuracy (MDA) was used to measure the relative abundance of each bacterium on day 8 at the species level in the predictive model. A heatmap depicts the comparison of bacteria in two groups filtered by random forest and 5-fold cross-validation (RFCV). (B) Relative abundance of seven taxa screened by random forest in the two groups on day 8. (C) RFCV models to predict biomarkers in the two groups on day 14. (D) Relative abundance of four taxa in the two groups on day 14. Statistical analysis was calculated with a two-tailed Student's *t* test. *, *P < *0.05; **, *P < *0.01, ***, *P < *0.001. Data are presented as the mean ± SEM. (E to G) Cooccurrence network maps capture the complexity of network interactions between gut microbiota and phenotypic data on day 8 (E) or 14 (F and G). Nodes are colored according to the phylum they belong to. Edges are estimated by Spearman's rank correlation coefficient, a red line between nodes represents a positive correlation, and a blue line represents a negative correlation (*P < *0.05).

It is clear that during the recovery phase of gut inflammation, probiotics have a superior ability to attenuate the disease phenotype and reshape the gut microenvironment in a DSS-induced colitis mouse model. Next, to robustly identify the microbial biomarker during the recovery phase of gut inflammation, random forest analysis and 10 trials of 5-fold cross-validation (RFCV) were performed for the probiotic consortia group and the control group on days 8 and 14, respectively (Fig. 4A and C; Fig. S3A and B). Analysis of the model’s union revealed significantly different bacteria in the two groups, with seven strains on day 8 and four strains on day 14. Specifically, the relative abundances of *Parasutterella Burkholderiales* and *Lactobacillaceae Lactobacillus* were significantly decreased and the relative abundances of *Muribaculaceae Muribaculaceae*, *Oscillospirales* UCG.005, *Dubosiella Firmicutes*, *Tissierellales Anaerovoracaceae*, and *Clostridia Clostridia* were increased in the probiotic consortia group compared with the control group on day 8 (Fig. 4B). Additionally, compared with the control group on day 14, the relative abundance of *Bacteroidales Tannerellaceae* and *Rikenellaceae Rikenellaceae* was significantly lower and the relative abundances of *Bacteroidaceae Bacteroides* and *Lachnospiraceae Ruminococcus* were significantly higher after the probiotic consortia treatment (Fig. 4D). Notably, except for *Lactobacillaceae Lactobacillus*, *Dubosiella Firmicutes*, and *Rikenellaceae Rikenellaceae*, the remaining eight differential strains were not significantly different in the single-strain treatment group and the control group (Fig. S3C and D). All of these data demonstrate that probiotic consortia treatment is very likely to exert beneficial effects by exhibiting superior strain modulation.

Additionally, the cooccurrence network maps were plotted to explore the interactions among gut flora and disease phenotypes for both groups (Fig. 4E to G). Notably, *Bacteroidaceae Bacteroides*, *Muribaculaceae Muribaculaceae*, *Bacteroidales Tannerellaceae*, and *Parasutterella Burkholderiales* were found in all three networks, and on day 8, they showed more complex interactive network relationships (Fig. S3E to G). Meanwhile, KEGG-based PICRUSt functional prediction showed that probiotic consortia treatment may protect intestinal epithelial cells by decreasing the pathway enrichment for bacterial invasion of epithelial cells and reducing the enrichment of riboflavin, cysteine, and methionine metabolism and other oxidative stress pathways (Fig. S4). It is worth noting that bacterial pathogenesis, such as lipopolysaccharide biosynthesis and flagellar assembly, is also reduced in the probiotic consortia group.

### The mixed metabolites attenuated symptoms in a DSS-induced colitis mouse model.

**FIG 5:**
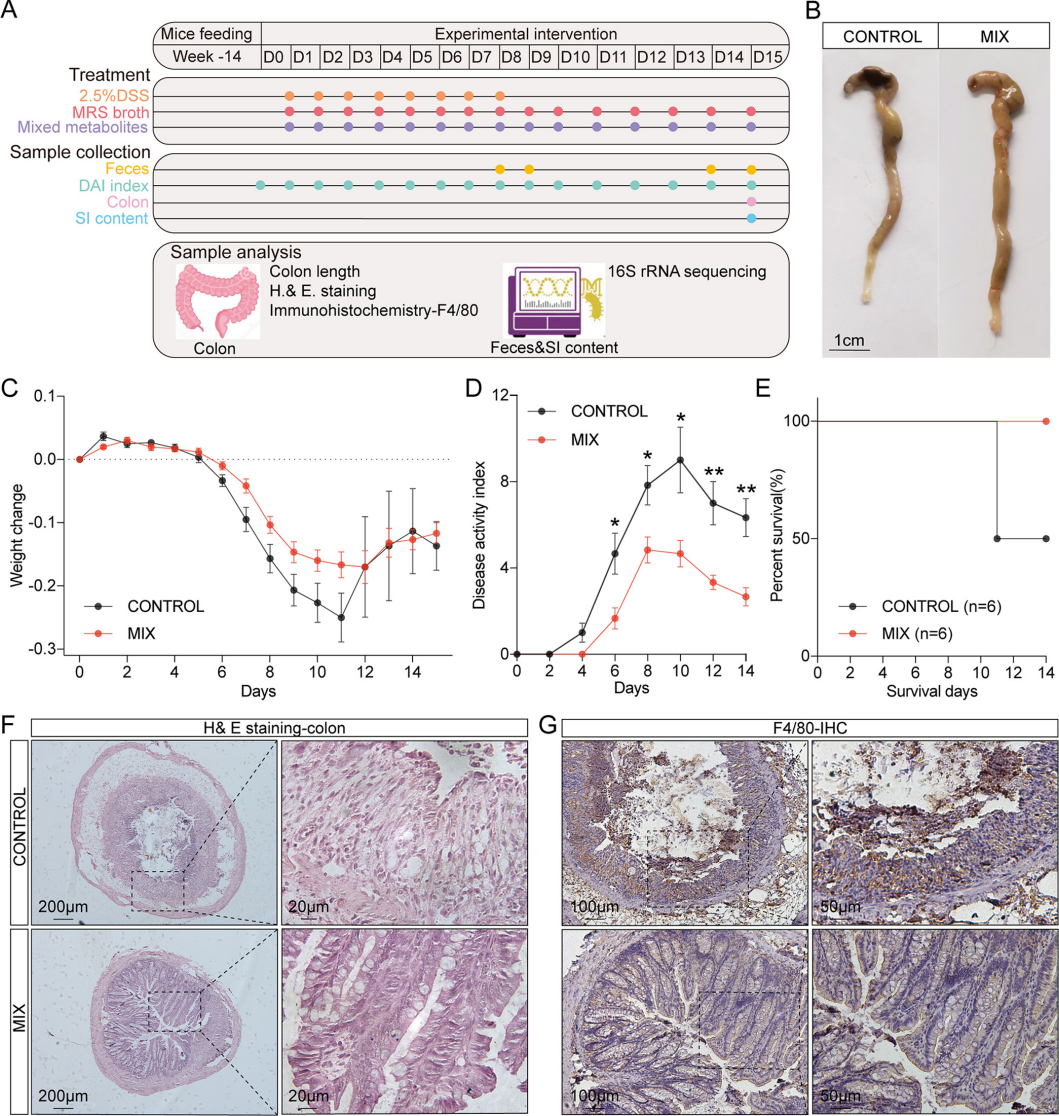
Mixed metabolites reduced colonic damage of the colitis mouse model. (A) Experimental design for mixed-metabolite treatment on DSS-induced colitis in mice. (B) Macroscopic pictures of colons. (C) Weight loss (*n* = 6). (D) DAI (*n* = 6). (E) Survival curve (*n* = 6). (F and G) Representative microscopic image of H&E staining (F) and F4/80 IHC (G). Statistics were calculated with a two-tailed Student's *t* test. *, *P < *0.01; **, *P < *0.01 (C and D). Data are presented as the mean ± SEM.

In addition to the colonization of healthy bacteria or probiotics on the mucus throughout the whole intestine, these beneficial bacteria are able to produce numerous metabolites, such as SCFAs, lipids, proteins, carbohydrates, and vitamins, some of which have an antagonistic intervention with pathogenic microbes in adhesion to the gut epithelial cells (44). Furthermore, they also regulate, synthesize, and degrade a wide range of biologically active metabolites, most of which are important signaling molecules for the intestine and other organs, promoting health through direct or indirect metabolic pathways (45). To further investigate the impact of metabolites on colitis, crude probiotic fermentation metabolites were administered to a DSS-induced colitis mouse model, which included only mixed-metabolite and control groups (Fig. 5A), because mixed strains were more effective than single strains in the probiotic consortia intervention experiment. The preparation of the mixed metabolites was processed with the suspension of the five strains of probiotics mixed in equal proportions. The suspension was centrifuged to initially obtain the supernatant and then aseptically purified by microporous filtration to extract the crude probiotic fermentation metabolites, which included mainly fatty acids and bile acids and are named postbiotics (46).

In addition, not only were the colonic feces collected on days 8 and 14, but the content of the small intestine (SI) was also collected on day 14 (Fig. 5A). Phenotypic observation indicated that mice in the control group had shorter colons and a steeper trend of weight loss with DSS administration (Fig. 5B and C; Fig. S5A and B), while the mix group with gavage of mixed metabolites showed significantly reduced DAI scores and improved survival rate compared with the control group (Fig. 5D and E; Fig. S5C). In addition, in the control group, the H&E-stained colon tissue displayed crypt loss, incomplete structure, mucosal necrosis, inflammatory cell infiltration, and goblet cell depletion triggered by DSS treatment (Fig. 5F), which led to a higher histological colitis score than that of the mix group (Fig. S5D). In contrast, the mixed metabolites inhibited DSS-induced histopathological injury by resulting in an intact colonic structure and less mucosal damage. The expression of F4/80 between the two groups also corresponded to H&E staining; specifically, the mix group exhibited less macrophage infiltration than the control group (Fig. 5G).

### The microecology of the small intestine but not the colon was modulated by mixed-metabolite intervention.

**FIG 6:**
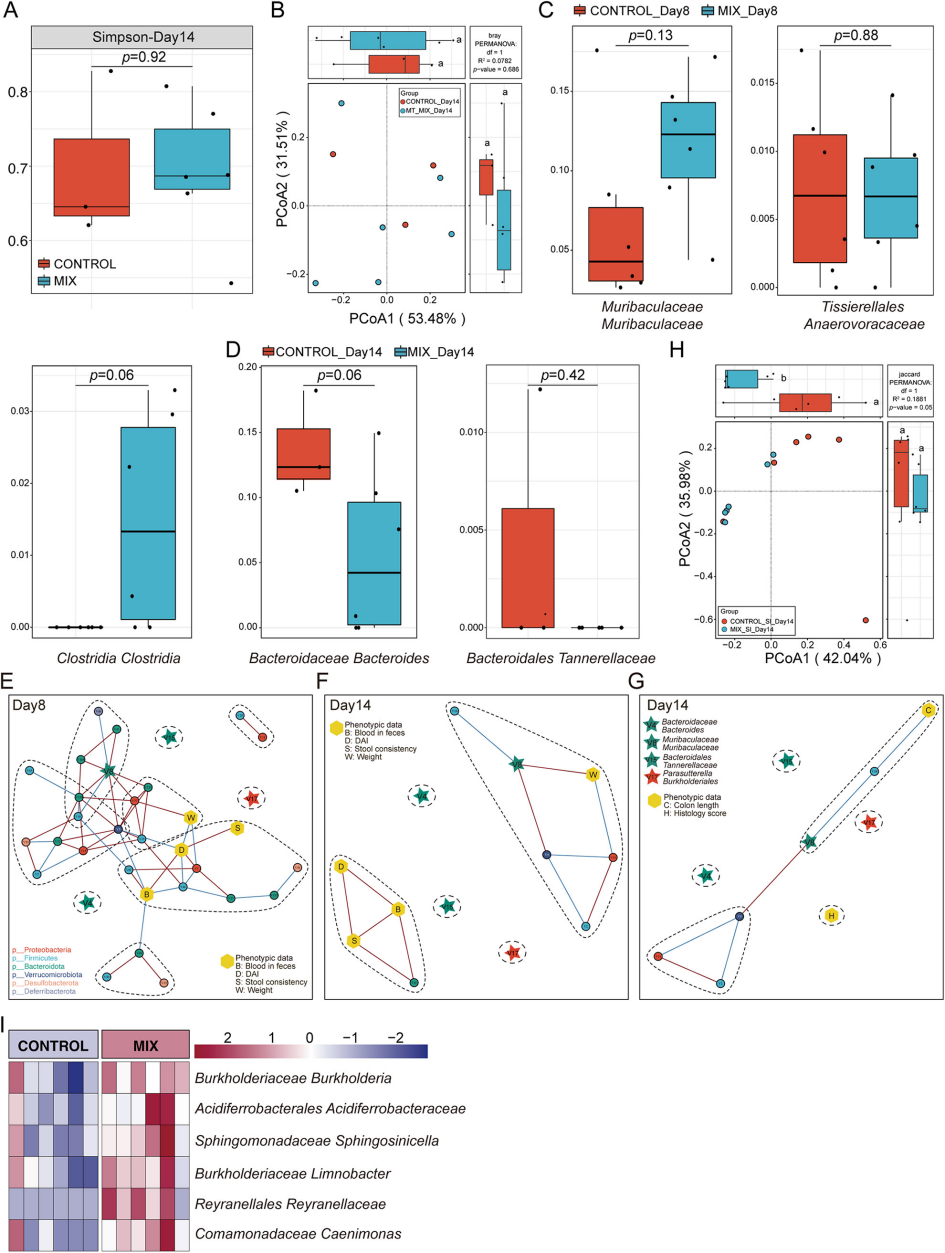
Mixed-metabolite intervention in the DSS-induced colitis mouse model was inferior to mixed-strain intervention in terms of specific regulation of the gut microbiota. (A) α-Diversity (Simpson) in the mixed-metabolite and control groups for colon feces on day 14. (B) β-Diversity in the mixed-metabolite and control groups for colon feces on day 14. (C and D) Relative abundance of key bacteria screened by the mixed-strain intervention in the mixed-metabolite and control groups in colon feces on day 8 or 14. (E to G) Cooccurrence network map between key signature bacteria and phenotypic data for colon fecal samples on day 8 (E) or 14 (F and G). Nodes are colored according to the phylum to which they belong. Edges are estimated by Spearman's rank correlation coefficient, a red line between nodes represents a positive correlation, and a blue line represents a negative correlation (*P < *0.05). (H) β-Diversity between mixed-metabolite group and control group in small intestinal contents on day 14. (I) Heatmap indicating bacteria with significant differences (*P < *0.05) in small intestinal contents on day 14. Statistics were calculated with a two-tailed Student's *t* test. Data are presented as the mean ± SEM.

The mixed-metabolite treatment was as effective as that of the mixed-strain intervention. Whether metabolite treatment also modulates the diversity, structure, and function of gut microbiota was also assessed. Between the control and mixed-metabolite groups on day 14, the α- and β-diversities of colon fecal samples were not significantly different (Fig. 6A and B), so the relative abundance of key bacterial biomarkers, which were key bacterial biomarkers identified in the probiotic consortia-treated group (e.g., *Parasutterella Burkholderiales*, *Oscillospirales UCG.005*, and *Lachnospiraceae Ruminococcus*), can barely be seen in either group (Fig. 6C and D). Meanwhile, the cooccurrence network maps revealed that *Bacteroidaceae Bacteroides*, *Bacteroidales Tannerellaceae*, and *Parasutterella Burkholderiales* all exhibited isolated points in the network (Fig. 6E to G). Although *Muribaculaceae Muribaculaceae* was in an important node position in the network (Fig. S6A to C), a comparison of the mix and control groups in the strain and metabolite intervention experiments revealed that only the probiotic consortia significantly altered the relative abundance of *Muribaculaceae Muribaculaceae* (Fig. S6D).

The above results confirmed that mixed-metabolite treatment has less effect on the modulation of gut flora in the colon. In addition, it is well known that metabolites from probiotics, such as SCFAs, amino acid, and vitamins, are absorbed in the small intestine. For example, oral administration of butyrate significantly improved butyrate levels in the blood and small intestine but not in the colon (47, 48). Therefore, we hypothesized that mixed metabolites may be more potent in influencing bacterial diversity and function in the small intestine. To test our hypothesis, we explored 16S rRNA gene sequencing of the small intestinal contents collected on day 14 in both the mixed-metabolite and control groups. Although there was no significant difference in α-diversity between the two groups, β-diversity indicated that the structure of the intestinal flora in the mix group was different from that in the control group (Fig. S6E; Fig. 6H). The heat map analysis of the core flora in the small intestinal contents also suggested significantly different flora between the two groups (Fig. 6I). These results support the hypothesis that mixed metabolites are likely to alter the bacterial ecology in the small intestine and that both metabolites and bacteria in the small intestine work cooperatively during the recovery phase of gut inflammation.

### Identification of microbial biomarkers in the small intestine is associated with mixed-metabolite intervention based on random forest analysis.

Under the administration of mixed metabolites, the flora composition of the small intestine was significantly different between the two groups (Fig. S7A). RFCV was performed to further investigate the intestinal flora that exerted a key impact under metabolite intervention (Fig. S7B and C), and the analysis of the model’s union revealed three taxa of bacteria. The relative abundances of *Acidiferrobacterales Acidiferrobacteraceae*, *Sphingomonadaceae Sphingosinicella*, and *Reyranellaceae Reyranella* were significantly increased in the mix group compared with the control group (Fig. S7D). Next, cooccurrence network analysis was conducted to explore the interactions between intestinal flora and disease phenotypes between the two groups (Fig. S7E and F). Although body weight and colon length were not tightly connected in the network, three strains occupied major nodes (Fig. S7G and H). In other words, these microbial biomarkers played important roles in remodeling the bacterial microenvironment in the small intestine of the DSS-induced colitis mouse model under the mixed-metabolite intervention.

## DISCUSSION

The aim of our study was to elucidate the comprehensive effect of probiotic consortia and their metabolites on the recovery of gut inflammation in a DSS-induced colitis mouse model. The alleviation was attributed mainly to the protection of the intestinal structure, function, and microenvironment. These results provide valuable insights into alternative therapeutic approaches for colonic inflammation using functional probiotics, paving the way for the application of probiotic consortia in IBD.

In the current study, we observed that the probiotic consortia ameliorated the symptoms of mice with DSS-induced colitis, as evidenced by a reduction in shortened colon length, weight loss, DAI score, mucosal loss, and inflammatory cell infiltration, which are in accordance with previous studies (37, 49–51). Additionally, IHC revealed a reduction in F4/80^+^ macrophages in the probiotic consortia group. Dysregulation of macrophages leads to inflammation and loss of tolerance to gut microbiota (52), and the total number of macrophages is increased as luminal contents enter the mucosa through the disrupted intestinal barrier (53). Notably, the single-strain group was not as effective as the probiotic consortia group in reducing body weight changes, DAI, and colonic histological scores. In addition, in the metabolite intervention experiment, the mix group did not differ significantly from the control group in terms of histological scores, which suggests that the probiotic consortia had a better intervention effect on the disease phenotype of the colitis mouse model than the mixed metabolites. The reason may be related to the fact that after entering the gut, in addition to interacting directly with mucosal epithelial cells, the bacterial strains can also directly suppress inflammation by affecting the overall microbial network (54).

Our results validated that the probiotic consortia restored microecological dysregulation in colitis mice on both day 8 of colonic inflammation progression and day 14 of the recovery phase. The α- and β-diversities reveal that the structural composition of the intestinal microbiota of mice in the mix group showed significant alterations compared with the control group, demonstrating that the altered microbiota may have an essential role in mitigating the progression of colitis, in accordance with the studies of Sun et al. and Wang et al. (51, 55). It was obvious that the recovery of gut microbiota by the single strain mainly occurred on day 14, which was not as effective as the probiotic consortia. Furthermore, the mixed metabolites did not alter the microbial community of the colon, but they had a significant influence on the microbial ecology of the SI. Patients with IBD have been found to have malabsorption and micronutrient deficiencies, which may be affiliated with microecological dysregulation in the SI, whereas metabolites are readily absorbed by the intestine after entering the host and can come into direct contact with small intestinal epithelial cells, increasing utilization (56–58). The metabolites also have a favorable regulatory effect on the intestinal flora due to the presence of bacteriocins with antimicrobial activity (46). Therefore, it is logical to speculate that metabolites may indirectly exert anti-inflammatory effects by further regulating the microbial community after absorption throughout the gastrointestinal tract.

Considering the effects of altered microbiota on colitis, we suspect that probiotic consortia interventions may take effect via potential microbial biomarkers in the intestine. Based on this idea, 11 characteristic bacterial taxa were identified by RFCV. Among them, the single strains significantly affected only three taxa, namely, *Lactobacillaceae Lactobacillus*, *Dubosiella Firmicutes*, and *Rikenellaceae Rikenellaceae*, indicating that single strains have a relatively weaker effect on the gut microbiota. The probiotic consortia had specific regulatory effects on the remaining eight taxa. Then, the analysis revealed that six taxa were dominant in the mix group compared with the control group, including *Muribaculaceae Muribaculaceae*, *Bacteroidaceae Bacteroides*, *Oscillospirales* UCG.005, *Lachnospiraceae Ruminococcus*, *Tissierellales Anaerovoracaceae*, and *Clostridia Clostridia*. *Muribaculaceae* were investigated for their potential benefits in reducing inflammation, suppressing harmful bacteria, and promoting anticancer immunity (59). *Bacteroides* is a prime candidate for the next generation of probiotics, producing SCFAs and sphingolipids that promote intestinal barrier function and modulate the immune response to repair intestinal damage (60, 61). Of the other four taxa, *Oscillospirales* UCG.005 and *Lachnospiraceae Ruminococcus* are butyrate-producing taxa and exhibit low abundance in patients with UC and CRC (62). The remaining two taxa belong to *Clostridia* and boost the proliferation and differentiation of Treg cells to attenuate IBD and allergic diarrhea (63). In contrast, the control group had a significantly increased relative abundance of *Parasutterella Burkholderiales* and *Bacteroidales Tannerellaceae* due to the DSS treatment. This is in accordance with previous studies (64). *Parasutterella Burkholderiales* is closely correlated with chronic inflammation in the colon, and its level is elevated in IBD (65, 66). Of importance, the relative abundance of these eight taxa in the metabolite intervention experiment did not show significant differences between the mix and control groups, further suggesting that mixed metabolites are less capable of regulating the colonic microbiota. Thus, we speculated that metabolite administration may affect the microbial ecology in the small intestine. It was shown that the relative abundance of *Acidiferrobacterales Acidiferrobacteraceae*, *Sphingomonadaceae Sphingosinicella*, and *Reyranellaceae Reyranella* was significantly lower in the control group, confirming the previous hypothesis that metabolites may act by maintaining the balance of the microenvironment in the small intestine.

Although it is generally accepted that IBD correlates with changes in the composition and metabolism of the gut microbiota, a direct causal relationship between dysbiosis and IBD has not been clearly identified in humans (27). To date, few studies have focused on the association between gut microbiota and IBD phenotype. The RDA of the current study provides powerful evidence to support the correlation between the microbial data and the colitis phenotypic data in the probiotic consortia intervention experiment, where species-level core microbial data were highly interpretable for relevant phenotypic data involving both disease severity and colonic pathology. To gain further understanding of whether microbial markers are also associated with phenotypic data, we generated cooccurrence networks, and in the postbiotic consortia intervention experiment, it was easier to note that *Bacteroidaceae Bacteroides*, *Muribaculaceae Muribaculaceae*, *Bacteroidales Tannerellaceae*, and *Parasutterella Burkholderiales* were all present in particular communities and showed a stronger and broader network interaction complexity at day 8. However, in the metabolite intervention experiments, the microbial markers were not strongly attached to the phenotypic data, and many were independent taxonomic units rather than symbiotic communities. Intriguingly, *Muribaculaceae Muribaculaceae* occupied a central position in the cooccurrence networks in both intervention experiments, suggesting the critical importance of these bacteria for the overall microbiota and disease phenotype cross talk. In conclusion, the probiotic consortia could alleviate gut inflammation in a colitis mouse model and restore dysfunction.

### Conclusion.

In the present study, we demonstrate that probiotic consortia treatment attenuates gut inflammatory symptoms of colitis by modulating the structure and composition of gut microbiota, with a stronger association between gut flora and colitis phenotype. The findings from the current animal model provide new insights into the maintenance of intestinal health with administration of probiotic consortia and will facilitate the development of probiotic-based therapeutic strategies for IBD and other gut inflammatory diseases in clinical practice.

### Limitations of the study.

There are several limitations of the current study. First, in future studies, we will apply metagenomics to determine the bacterial composition alteration at the species level. Second, the expected inflammatory pathways induced by changes in gut microorganisms need to be investigated further to identify appropriate targets for therapeutic development. Third, the purification and identification of core components that act through mixed-metabolite intervention have not yet been clarified, and subsequent metabolomic analysis is needed.

## MATERIALS AND METHODS

### Animals.

Male *C57BL/6J* mice were purchased from the Experimental Animal Center of Guangzhou University of Chinese Medicine and raised in a specific-pathogen-free (SPF) animal facility at the Institute of Microbiology of the Guangdong Academy of Sciences. The animal facility was maintained with 12-h-light/12-h-dark cycles and was temperature and humidity controlled, and all experimental animals were provided *ad libitum* access to food and water. All animal operations and procedures were approved by the Animal Protection and Utilization Committee of the Institute of Microbiology, Guangdong Academy of Sciences (permission no. SCXK [Guangdong] 2018-0034).

This intervention study was designed to administer probiotic strains or probiotic fermentation metabolites in a DSS-induced colitis mouse model. The probiotic strain treatment was administered to 7 groups. These groups were named the Lactobacillus reuteri PLBK1 group (LR1), Lactobacillus reuteri PLBK2 group (LR2), Lactobacillus gasseri PLBK3 group (LG), Lactobacillus acidophilus PLBK4 group (LA), Bifidobacterium lactis PLBK5 group (BL), the probiotic consortia (mix) group with the five strains mixed in equal proportions, and the control group (control) with a PBS gavage containing 30% glycerol. The probiotic metabolite experiment consisted of two groups: the mix group of five probiotic fermentation metabolites in equal ratios and the control group (control) with sugar-free MRS medium.

### Establishment of the dextran sulfate sodium (DSS)-induced colitis mouse model.

C57BL/6J mice were given 2.5% DSS (lot no. S2839; molecular weight, 36,000 to 50,000 Da; MP Biomedical, LLC, Solon, OH, USA) in sterile water for 7 days to establish a colitis mouse model, followed by recovery with regular drinking water for an additional 7 days. The probiotic strains or metabolites were administered via an intragastric administration of 200 μL each time throughout the experimental period, once a day for probiotic strains and three times a day for metabolites. The mice were sacrificed after the fecal samples, colon tissue, and intestinal epithelial scrapings were collected.

### Bacterial strain culture and counting.

Lactobacillus reuteri PLBK1, Lactobacillus reuteri PLBK2, Lactobacillus gasseri PLBK3, Lactobacillus acidophilus PLBK4, and Bifidobacterium lactis PLBK5 were originally isolated from healthy Chinese feces, deposited in Guangdong Provincial Key Laboratory of Microbial Culture Collection and Application, and assigned a designated strain number. These probiotic strains are shared by ProBioCare Biotechnology Co., Ltd. The bacteria were cultured in MRS broth (HB0384-1; Hopebio, Qingdao, China) at 37°C in an aerobic incubator for 48 h. The suspension was centrifuged at 4°C for 10 min at 3,000 relative centrifugal force (rcf), and the precipitant was then resuspended in sterile PBS and 30% glycerol to a final concentration of 1 × 10^9^ CFU/200 μL and stored at −80°C. For the metabolite intervention experiment, the cultured mixed-strain suspension was centrifuged at 4°C for 30 min at 3,000 rcf to collect the supernatant, followed by filtration through a 0.22-μm filter (lot no. 210518-228; Biofil, Guangdong, China) to sterilize the metabolite supernatant. The supernatant containing the metabolites was divided and stored at 4°C.

### Evaluation of colonic inflammation (DAI).

Inflammation of colitis in each group was assessed by the disease activity index (DAI), which is a comprehensive assessment of (i) body weight loss (%), (ii) stool consistency, and (iii) the amount of blood in feces. The change in body weight relative to initial body weight (day 0) was recorded every day, and the specific calculation is shown in Table S1 in the supplemental material. The DAI is the sum of all three data points, with values ranging from 0 to 12 (67).

### H&E staining.

The colon tissues were collected and fixed in 4% paraformaldehyde at room temperature for 48 h, dehydrated, embedded in paraffin, and sectioned into 4-μm-thick sections by a slicer. Finally, the colon tissue sections were stained with hematoxylin and eosin (H&E) as described previously (18, 68).

### Histological score.

The images of H&E-stained colon tissues were captured by a research-grade upright light microscope (Leica DM6B), and the pathological score was calculated in a blind fashion based on the following comprehensive score criteria: (i) epithelial loss, (i) crypt damage, (iii) depletion of goblet cells, and (iv) infiltration of inflammatory cells (69), as shown in Table S2.

### Immunohistochemistry.

Fixed and sectioned colon tissues were dewaxed at 65°C and rehydrated with xylene (2 times for 10 min each), anhydrous alcohol (2 times for 10 min each), 95% alcohol (5 min), 70% alcohol (5 min), 50% alcohol (5 min), and distilled water (to rinse the slides). Antigen retrieval was performed by placing the slides in a boiled antigen retrieval solution at 100°C for 1 h, followed by antigen repair by incubation with 3% H_2_O_2_ at room temperature (RT) for 5 to 10 min. Blocking buffer containing 3% bovine serum albumin (BSA), 5% goat serum, and 0.5% Triton 100 was applied to each section for at least 1 h of incubation at RT. Then, each section was incubated with primary (overnight at 4°C, F4/80; lot no. LS194062; Servicebio, Wuhan, China) and secondary (RT in the dark, HRP-conjugated 2′ antibody) antibody diluted in blocking buffer. Sections were washed in PBS and incubated with 3,3-*N*-diaminobenzidine tetrahydrochloride (DAB), followed by imaging with an upright light microscope (Leica DM6B) as described previously (70).

### DNA extraction from feces, sequencing library construction, and 16S rRNA sequencing.

DNA from mouse feces was extracted by utilizing a MoBio PowerSoil DNA extraction kit (Qiagen, USA), and the concentration was measured with a nanodrop (ThermoFisher, USA). Fifty nanograms of DNA was used for 16S rRNA sequencing library construction using Q5 high-fidelity DNA polymerase (NEB), targeting the V3-V4 region of the bacterial 16S rRNA gene (forward primer, 5′-CCTACGGGNGGCWGCAG-3′; reverse primer, 5′-GACTACHVGGGTATCTAATCC-3′), followed by purification with AMPure XP (Beckman).

### 16S rRNA amplicon sequencing and bioinformatics statistics.

A standard QIIME 2 pipeline was approved, and high-quality amplicon sequence variants (ASVs) were obtained by the DADA2 algorithm (71, 72). The taxonomy profile analysis was performed against the Silva database (73) and transformed into relative abundance at the phylum, class, order, family, genus, and species levels.

Microbial data indicating a relative abundance less than 0.001 or an attendance rate less than 70% in all groups were filtered to obtain core bacterial taxa for further analysis. α- and β-diversity analyses, cooccurrence analysis, structure plot, and random forest model were performed using the R package EasyMicroPlot (74).

### Statistical analysis.

The statistical details of all experiments and the sample numbers are described in the figure legend of each figure. The results are presented as the mean ± standard error of the mean (SEM). For significant comparisons between two groups, a two-tailed, unpaired Student's *t* test was used. Significance among multiple groups was tested using a one-way analysis of variance (ANOVA), followed by Tukey’s and the least significant difference (LSD) *post hoc* test. Statistical significance is described in the figure legends as follows: *, *P < *0.05; **, *P < *0.01; ***, *P < *0.001.

### Data availability.

All raw sequence data have been uploaded into the NMDC and NCBI databases under accession numbers NMDC10018004 and PRJNA809042, respectively.
