# Supplementary material for: Probiotic Consortia and Their Metabolites Ameliorate the Symptoms of Inflammatory Bowel Diseases in a Colitis Mouse Model
Source: Microbiol Spectr. 2022 Jun 22;10(4):e00657-22. doi: 10.1128/spectrum.00657-22 (PMC9430814; doi:10.1128/spectrum.00657-22)
Supplement: Supplemental file 2 — Supplemental material. Download spectrum.00657-22-s0002.pdf, PDF file, 2.5 MB [file spectrum.00657-22-s0002.pdf]

Probiotic consortia and their metabolites ameliorate the symptoms of inflammatory bowel diseases in a colitis mouse model

Limin Xu[1,2,#], Bingdong Liu[2,#], Liujing Huang[2,3,#], Ze Li[1,2,#], Yanbo Cheng[2], Ye Tian[2], Guihua Pan[2], Huijun Li[1] ,Yinlan Xu[1], Weidong Wu[1], Zongbin Cui[2], Liwei Xie[1,2,3,*]

Supplementary figures and legends

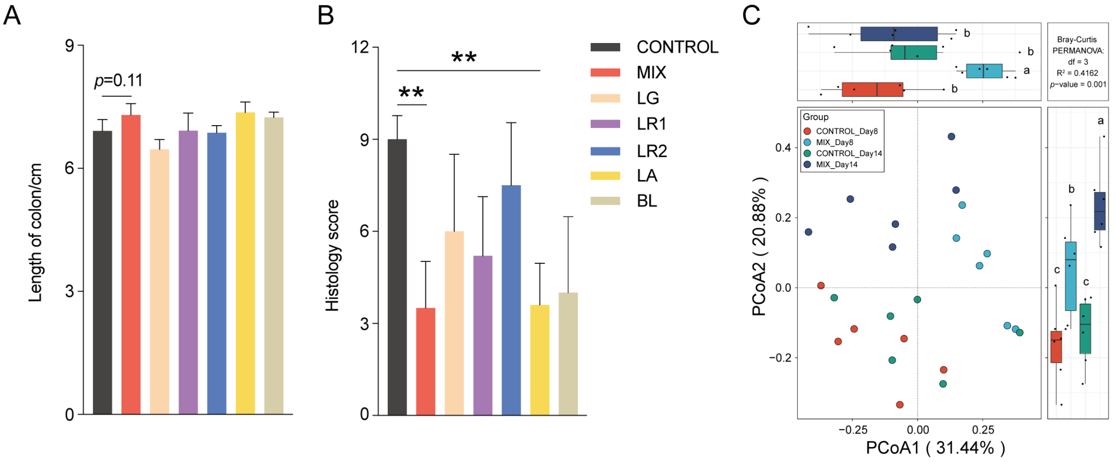

Figure S1: Related to Figure 1/2/3. (A) The lengths of colon from each group (n=6). (B) Histology scores of colons (n=6). (C) PCoA plot of the gut microbiota based on Bray-Curtis matrix on Days 8 and 14. Statistical analysis were calculated with one-way ANOVA. $^{**}p<0.01$. Data were presented as mean ± the standard error of the mean (SEM).

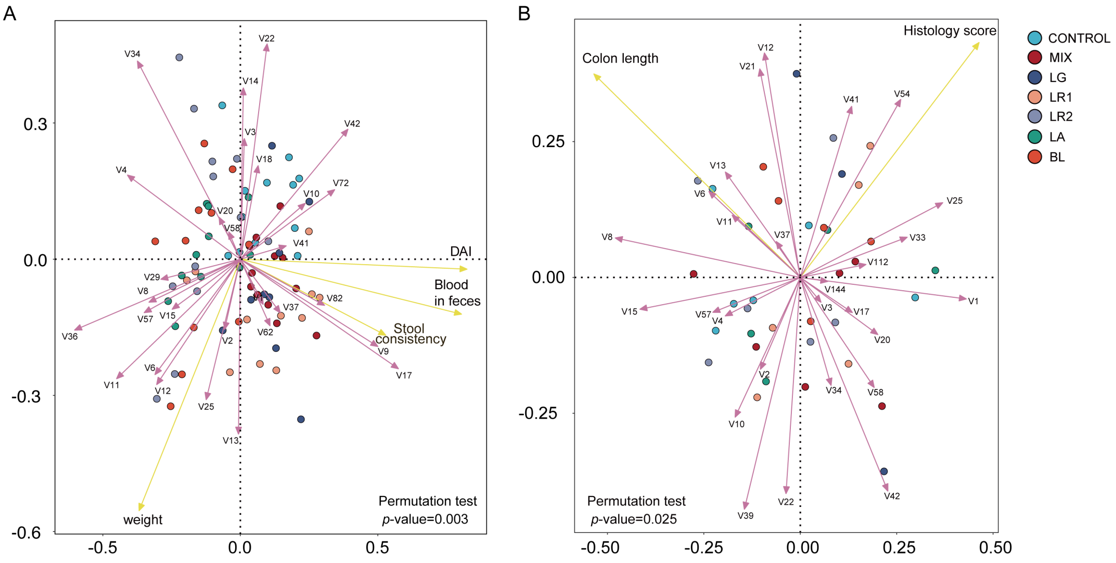

Figure S2. Redundancy analysis (RDA) plots. PCA revealed the correlations between bacterial community structures and weight, DAI, stool consistency, blood in feces (A) or colon length, histology score at species level (B). (A: V4: *Bacteroidaceae Bacteroides*: r^2=0.1152, p=0.016; V9: *Sutterellaceae Parasutterella*: r^2=0.1658, p=0.001; V17: *Parasutterella Burkholderiales*: r^2=0.2226, p=0.001; V36: *Muribaculaceae Bacteroidales*: r^2=0.2292, p=0.001; V34: *Firmicutes Bacilli*: r^2=0.1557, p=0.003; V42: *Mucispirillum schaedleri*: r^2=0.1228, p=0.009. B: V8: *Muribaculaceae Muribaculaceae*: r^2=0.2082, p=0.028; V39: *Enterobacteriaceae Klebsiella*: r^2=0.1823, p=0.028; V12: *Bacteroidales Muribaculaceae*: r^2=0.1673, p=0.045; V42: *Mucispirillum schaedleri*: r^2=0.1932, p=0.026). Each dot indicates one sample; bacteria are indicated by red arrows (The name of the bacterium can be found in Table S4), and phenotypic data are indicated by yellow arrows. The length of the arrow line represents the correlation between the bacterial distribution and the phenotypic data, the longer the arrow line, the higher the correlation. The angle between the arrow line and the ranking axis represents the correlation between them, the smaller

the angle, the higher the correlation, and vice versa, the lower the correlation. *$p<0.05$, **$p<0.01$, ***$p<0.001$. Statistical analyses were calculated with permutation test.

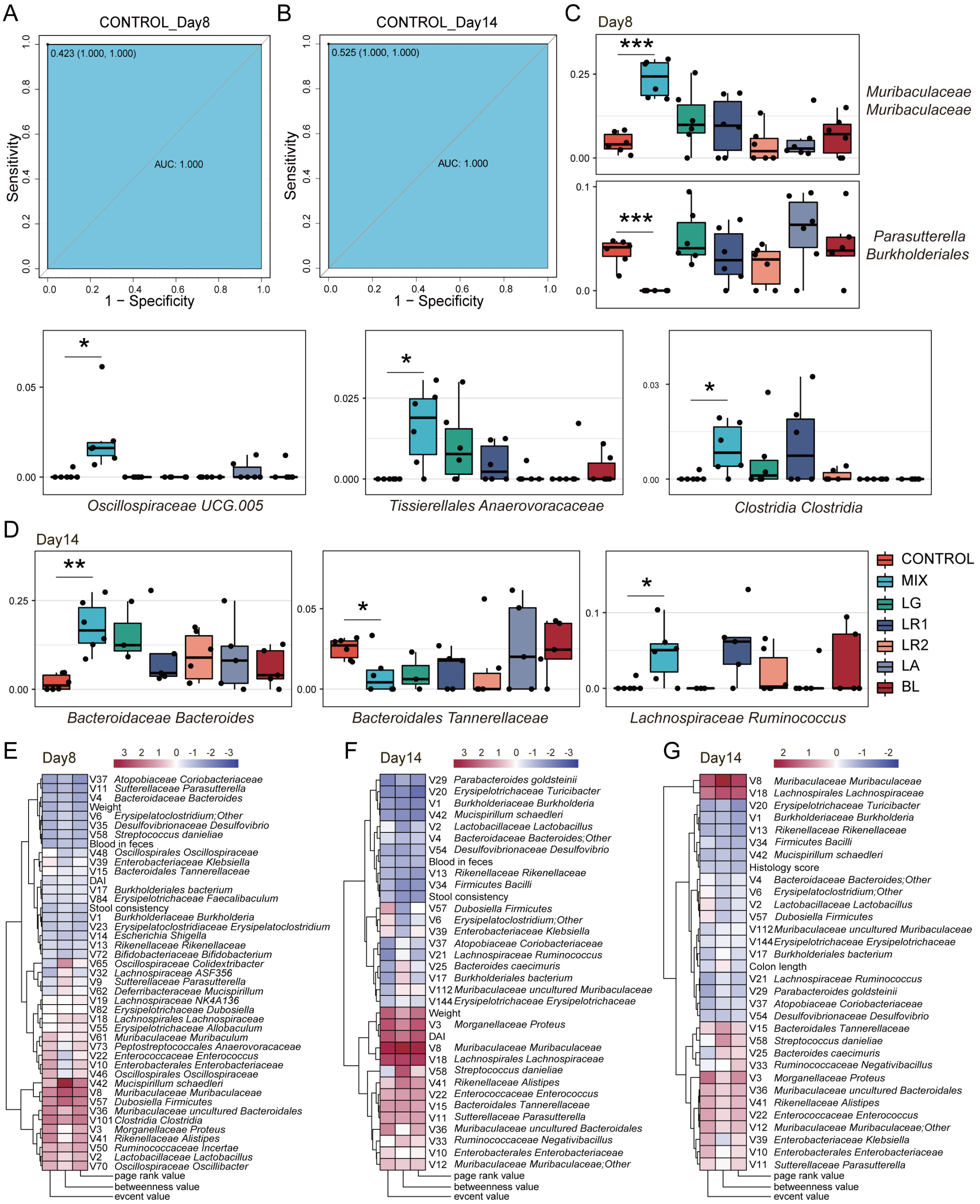

Figure S3: Related to Figure 4. (A-B) The curve of receiver operating characteristic was performed to test the prediction model. (C-D) The floating box plot indicated the relative abundance of key bacteria in seven groups at different times. *$p<0.05$, **$p<0.01$, ***$p<0.001$. Data is displayed as mean ± the standard error of the mean. (E-G) The values of page rank, betweenness and evcent algorithm were used to evaluate

the importance of the nodes representing the gut microbiota and phenotypic data in the co-occurrence network map.

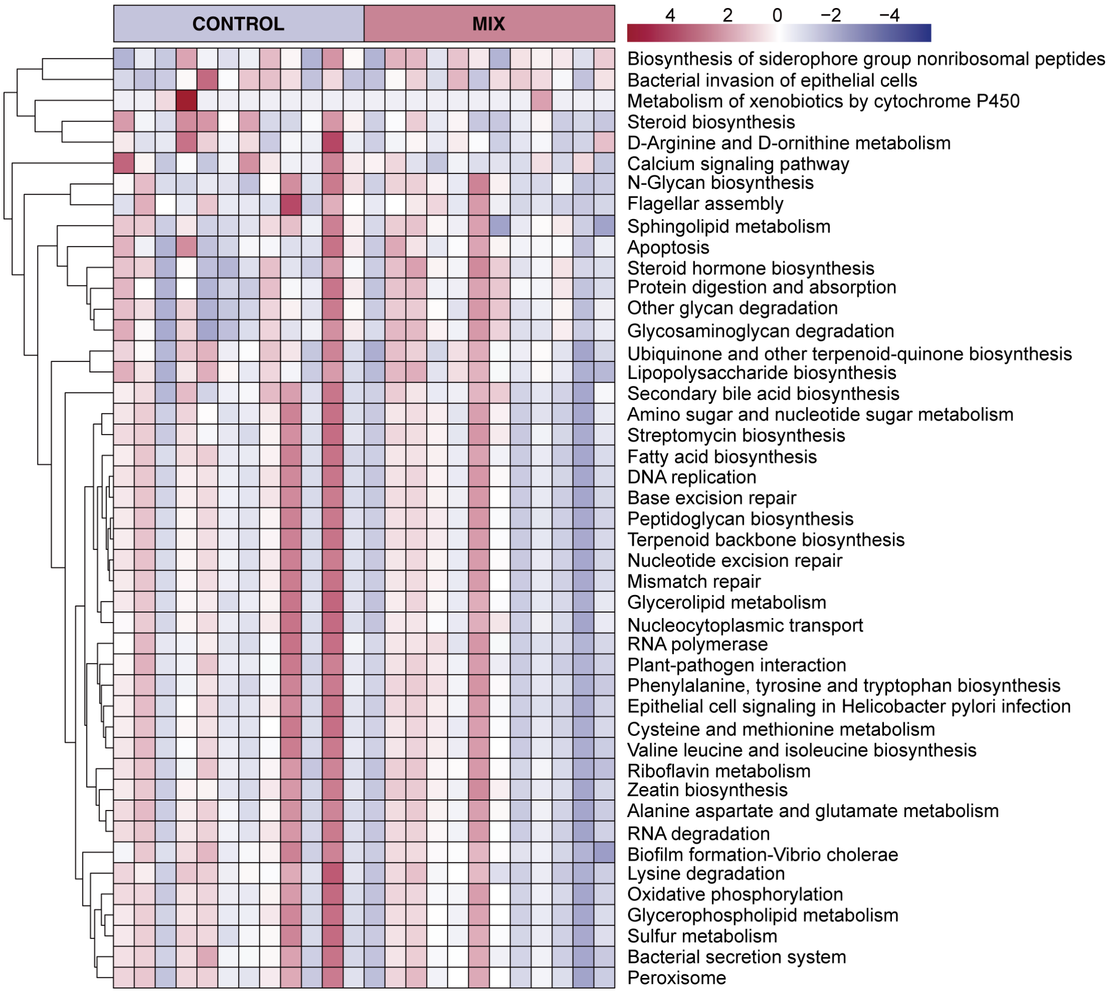

Figure S4. PICRUSt plot predicts significantly different metagenomic functions against o the KEGG database.

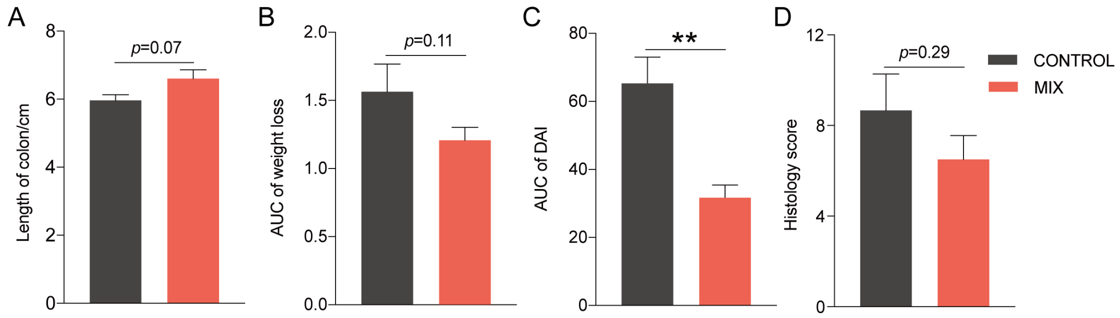

Figure S5: Related to Figure 5. (A) The lengths of colon from two groups (n=6). (B) AUC of weight loss (n=6). (C) AUC of DAI (n=6). (D) Histology scores of colons (n=6). Statistics were calculated with two-tailed Student's t-test. $^{**}p<0.01$. Data is presented as mean ± the standard error of the mean (SEM).

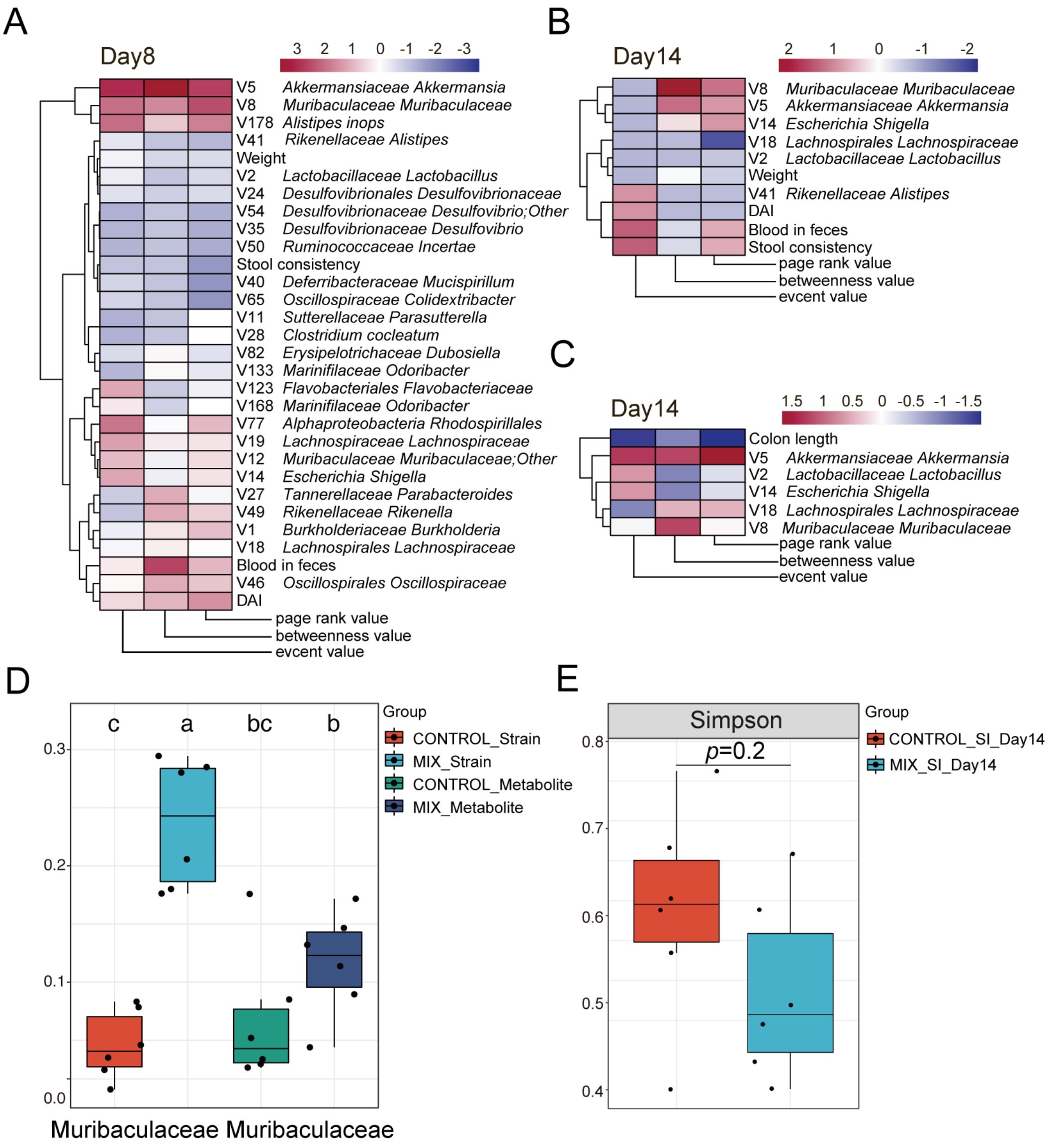

Figure S6: Related to Figure 6. (A-C) The values of page rank, betweenness and evcent algorithm were used to evaluate the importance of the nodes representing the gut microbiota and phenotypic data in the co-occurrence network map. (D) Relative abundance of *Muribaculaceae Muribaculaceae* in mix group and control group of mixed strains intervention and mixed metabolites intervention experiments for colon fecal on Day 8. (E) α-diversity between mix group and control group in small intestinal contents on Day14. Statistics were calculated with one-way ANOVA or two-tailed Student's t-test. Data is presented as mean ± the standard error of the mean (SEM).

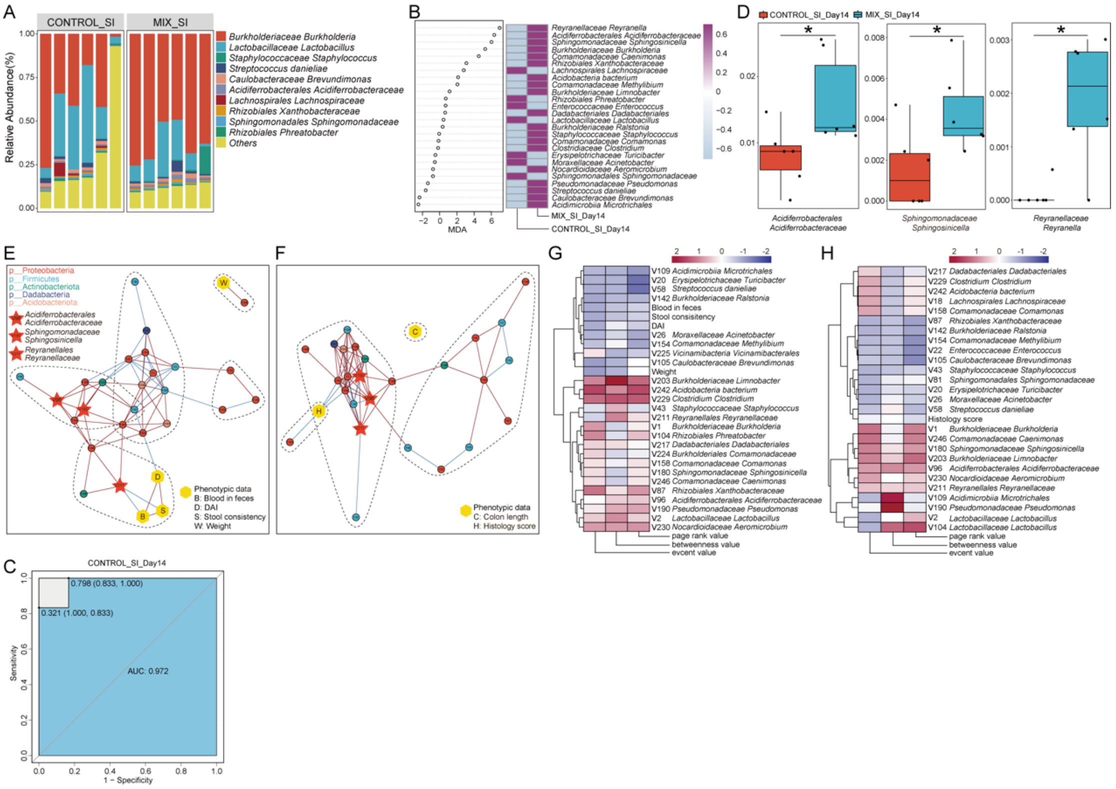

Figure S7. Identified essential bacteria involved in reshaping the intestinal microbiology in small intestine on Day 14. (A) Species-level structure plot of the top ten abundances. (B) MDA was used to measure the relative abundance of each bacterium at species level. The heatmap depicted the comparison of bacteria in two groups filtered by RFCV. (C) The curve of receiver operating characteristic was performed to test the prediction model. (D) The relative abundance of *Acidiferrobacterales Acidiferrobacteraceae*, *Sphingomonadaceae Sphingosinicella* and *Reyranellaceae Reyranella* between groups. Statistics were calculated with two-tailed Student's t-test. *p<0.05. Data is presented as mean ± the standard error of the mean (SEM). (E-F) The co-occurrence network maps describe the symbiotic relationship between intestinal bacteria and phenotypic data. Nodes are colored according to the phylum they belong to. Edges are estimated by Spearman's rank correlation coefficient, red line between nodes represents positive correlation and blue

line represents negative correlation ($p<0.05$). (G-H) The values of page rank, betweenness and evcent algorithm were used to evaluate the importance of the nodes representing the intestinal bacteria and phenotypic data in the co-occurrence network map.
